# Circulating ceramides and sphingomyelins and the risk of incident cardiovascular disease among people with diabetes: the strong heart study

**DOI:** 10.1186/s12933-022-01596-4

**Published:** 2022-08-30

**Authors:** Paul N. Jensen, Amanda M. Fretts, Andrew N. Hoofnagle, Barbara McKnight, Barbara V. Howard, Jason G. Umans, Colleen M. Sitlani, David S. Siscovick, Irena B. King, Nona Sotoodehnia, Rozenn N. Lemaitre

**Affiliations:** 1 Department of Medicine, University of Washington, 1730 Minor Ave, Suite 1360, Seattle, WA 98101 USA; 2 Department of Epidemiology, University of Washington, Seattle, WA USA; 3 Department of Laboratory Medicine, University of Washington, Seattle, WA USA; 4 Department of Biostatistics, University of Washington, Seattle, WA USA; 5 Cardiovascular Health Research Unit, University of Washington, Seattle, WA USA; 6 MedStar Health Research Institute, Hyattsville, MD USA; 7 Georgetown and Howard Universities Center for Clinical and Translational Science, Washington, DC USA; 8 New York Academy of Medicine, New York, NY USA; 9 Department of Internal Medicine, University of New Mexico, Albuquerque, NM USA

**Keywords:** Sphingolipids, Ceramides, Sphingomyelins, Cardiovascular disease, Epidemiology

## Abstract

**Background:**

Plasma ceramides and sphingomyelins have been independently linked to diabetes risk, glucose and insulin levels, and the risk of several cardiovascular (CVD) outcomes. However, whether individual ceramide and sphingomyelin species contribute to CVD risk among people with type 2 diabetes is uncertain. Our goal was to evaluate associations of 4 ceramide and 4 sphingomyelin species with incident CVD in a longitudinal population-based study among American Indians with diabetes.

**Methods:**

This analysis included participants with prevalent type 2 diabetes from two cohorts: a prospective cohort of 597 participants in the Strong Heart Family Study (116 incident CVD cases; mean age: 49 years; average length of follow-up: 14 years), and a nested case–control sample of 267 participants in the Strong Heart Study (78 cases of CVD and 189 controls; mean age: 61 years; average time until incident CVD in cases: 3.8 years). The average onset of diabetes was 7 years prior to sphingolipid measurement. Sphingolipid species were measured using liquid chromatography and mass spectrometry. Cox regression and logistic regression were used to assess associations of sphingolipid species with incident CVD; results were combined across cohorts using inverse-variance weighted meta-analysis.

**Results:**

There were 194 cases of incident CVD in the two cohorts. In meta-analysis of the 2 cohort results, higher plasma levels of Cer-16 (ceramide with acylated palmitic acid) were associated with higher CVD risk (HR per two-fold higher Cer-16: 1.85; 95% CI 1.05–3.25), and higher plasma levels of sphingomyelin species with a very long chain saturated fatty acid were associated with lower CVD risk (HR per two-fold higher SM-22: 0.48; 95% CI 0.26–0.87), although none of the associations met our pre-specified threshold for statistical significance of p = 0.006.

**Conclusions:**

While replication of the findings from the SHS in other populations is warranted, our findings add to a growing body of research suggesting that ceramides, in particular Cer-16, not only are associated with higher diabetes risk, but may also be associated with higher CVD risk after diabetes onset. We also find support for the hypothesis that sphingomyelins with a very long chain saturated fatty acid are associated with lower CVD risk among adults with type 2 diabetes.

**Supplementary Information:**

The online version contains supplementary material available at 10.1186/s12933-022-01596-4.

## Background

Diabetes is a major risk factor for cardiovascular disease (CVD) [1], contributing to up to one fifth of vascular disease incidence worldwide [2]. Randomized clinical trials have shown that normalization of glucose does not effectively reduce CVD risk among patients with diabetes [3–6]; novel, effective therapies to reduce the burden of CVD among people living with diabetes are urgently needed.

There is abundant evidence that ceramides are involved in diabetes in experimental studies [7, 8]. In addition, we have previously reported that plasma ceramides are associated with risk of incident type 2 diabetes in two large population studies, suggesting an involvement in diabetes in humans as well [9, 10].

Ceramide and its precursor sphingomyelin are part of the large sphingolipid family. At their core is a “sphingoid” backbone to which one fatty acid is acylated. There is increasing evidence that the length of the fatty acid acylated to backbone impacts the biological activities of ceramides and sphingomyelins. Cell and animal studies have suggested that Cer-16 promotes apoptosis, while longer chain ceramides (Cer-20, -22, and -24) appear to prevent apoptosis [11–15]. In addition, we have shown in the Cardiovascular Health Study that plasma levels of Cer-16 and SM-16 are associated with a higher risk of incident heart failure, atrial fibrillation, and mortality, while longer-chain ceramides and sphingomyelins are associated with a lower risk of each of these outcomes [16, 17]. In contrast, we have recently reported that both Cer-16 and its longer chain counterparts are associated with higher risk of incident diabetes, as well as with elevated fasting insulin and glucose levels [10, 18, 19]. Whether ceramide and sphingomyelin species influence cardiovascular disease risk among patients with prevalent type 2 diabetes has received limited attention.

Diabetes is common among American Indians (AIs), and managing its potential complications is a public health priority [20]. The purpose of this study was to assess whether ceramide and sphingomyelin with 16:0 are associated with higher risk of incident CVD, while ceramide and sphingomyelin species with 20:0, 22:0 and 24:0 are associated with lower risk of incident CVD among participants in the Strong Heart Study with prevalent type-2 diabetes.

## Methods

### Study population

The Strong Heart Study (SHS) and Strong Heart Family Study (SHFS) are population-based longitudinal studies of CVD in several American Indian communities in Arizona, North and South Dakota, and Oklahoma. Details of the study designs have been described previously [21]. Briefly, in 1989, SHS recruited a cohort of 4549 individuals who participated in clinical exams in 1989–91, 1993–95, and 1998–99. At the 1998–99 exam, 1st, 2nd, and 3rd degree relatives of SHS participants were invited to participate in a family study, which became the Strong Heart Family Study (SHFS). The SHFS included two examinations: 2001–03 and 2006–09. The institutional review boards from each Indian Health Service region and all communities approved the studies, and written informed consent was obtained from all participants at each exam.

Analyses were restricted to participants with prevalent type-2 diabetes at the time of their sphingolipid measurement. Prevalent diabetes was defined as a fasting plasma glucose ≥ 126 mg/dL or use of insulin or oral anti-diabetic medications at the relevant study exam; in SHS, where 2-h oral glucose tolerance tests were performed, we also identified prevalent diabetes as an oral glucose tolerance test ≥ 200 mg/dL [22].

Sphingolipids were measured prospectively at two timepoints in SHFS, allowing for the inclusion of 508 participants with prevalent diabetes at the 2001–03 exam and an additional 202 participants who were subsequently diagnosed with diabetes before or at the 2006–09 exam. Of these 710 participants, we excluded 96 who had a history of CVD at the time of their sphingolipid measurement and 17 who had no available follow-up CVD data, leaving 597 SHFS participants eligible for this analysis.

In SHS, we used a nested case–control design and samples from the 1993–95 exam. Selected cases and controls had prevalent diabetes and no history of CVD at the 1993–1995 exam. For cases, we randomly selected 100 participants out of 444 who subsequently developed CVD during follow-up in order to preserve scarce samples. We used risk set matching; for each case, we selected two age (within 5 years) and sex matched controls who had not developed incident CVD. Of the 300 SHS participants we sampled, 281 had sufficient blood samples and were able to have their sphingolipids measured; 14 participants from the Arizona site were excluded due to a lack of appropriate controls (13 cases and 1 control), leaving 267 participants (78 cases and 189 controls) in the analysis.

### Data collection

Each SHFS and SHS examination included a standardized personal interview, physical examination, and laboratory work-up. The data collection procedures were identical at all SHFS and SHS exams except for physical activity and diabetes ascertainment; these procedures have been described in detail previously [21, 23].

### Sphingolipid measurement

Sphingolipids were measured from blood samples collected at the 2001–03 and 2006–09 SHFS study examinations and the 1993–95 SHS study examination. The same collection protocols were used at each study exam; blood samples were collected after a 12-h fast and were stored at − 70 °C until analyzed. A detailed description of the laboratory procedures can be found elsewhere; briefly, plasma sphingolipids were quantified from baseline EDTA-blood samples using liquid chromatography–tandem mass spectrometry [24].

This analysis includes eight sphingolipids that carry a saturated fatty acid acylated to the sphingoïd backbone: ceramide and sphingomyelin with palmitic acid (16:0 [16 carbons, 0 double bonds]; Cer-16 and SM-16), with arachidic acid (20:0; Cer-20 and SM-20), with behenic acid (22:0; Cer-22 and SM-22), and with lignoceric acid (24:0; Cer-24 and SM-24).

### Incident cardiovascular disease

Incident CVD was defined as the first occurrence of myocardial infarction (MI; definite, probable, or fatal), ischemic stroke (definite or fatal), atherosclerotic cardiovascular disease (ASCVD; definite), or heart failure (HF; including fatal). Incident events in both SHFS and SHS were identified at each study examination and through subsequent surveillance of medical records and death certificates; after the 2006–09 examination and through December 2017, cases were ascertained through phone interviews with the participants and confirmed by medical record documentation. Stroke and HF were identified via chart review, MI was identified through chart reviews and/or evidence of MI by study visit ECG, and ASCVD was defined as incident MI, coronary heart disease (CHD), ischemic stroke, or abnormal study ECG with positive Rose Angina Questionnaire.

### Statistical analysis

In the SHFS cohort, Cox regression models were used to examine the associations of each sphingolipid with the risk of incident CVD. SHFS participants began accruing time-at-risk at the earliest study exam at which they had prevalent diabetes and measured sphingolipids (n = 434 from the 2001–03 exam, and n = 163 from the 2006–09 exam), and were followed to the earliest of: date of incident CVD, death, or loss to follow up. Because the SHFS is comprised of extended families, robust standard error estimates that account for clustering of risk factors among family members were used. In the SHS cohort, logistic regression with robust standard errors was used to evaluate the associations of plasma sphingolipids and incident CVD.

We used 3 sets of models to examine associations of sphingolipids with incident CVD. Model 1 (minimally-adjusted model) included terms for age, sex, and study site (SHFS only). Model 2 (multivariable model) additionally adjusted for education (years), smoking (never, former, current), alcohol consumption (never, former, current), physical activity (in SHFS: average number of pedometer measured steps per day; in SHS: metabolic equivalent task hours per week), body mass index (BMI; log-transformed), waist circumference, low density lipoprotein-cholesterol (LDL), systolic blood pressure, treated hypertension, duration of diabetes (log-transformed), type of diabetes medication used (insulin + oral hypoglycemic, insulin only, oral hypoglycemic only, or neither), and treated hyperlipidemia. Model 3 (sphingolipid adjusted model) included Model 2 with additional adjustment for one of the other species: Cer-16 and SM-16 models include adjustment for Cer-22 and SM-22, respectively; Cer-20, -22, -24, and SM-20, -22, and -24 models include adjustment for Cer-16 and SM-16, respectively. In both cohorts, adjustment covariate values were drawn from the same study exam at which the sphingolipids were measured. Sphingolipid exposures were log base-2 transformed, so the relative risk represents the associations with incident CVD per doubling of each of the sphingolipid species concentrations (µM), which is roughly equivalent to the difference between the 10th and 90th percentiles (Table 2). With the exception of study site in SHS, the same set of adjustment variables were used in each of the models for both SHFS and SHS cohorts.

As a secondary analysis, we evaluated associations of sphingolipids with incident MI, stroke, heart failure, and ASCVD within the SHFS cohort. These models followed the same analytic approach as the primary analysis.

We conducted a number of sensitivity analyses. To assess whether associations were robust to additional adjustment of high density lipoprotein-cholesterol (HDL), fibrinogen, or triglyceride levels, or of estimated glomerular filtration rate (eGFR), we repeated the multivariable analyses with additional terms for HDL-cholesterol, triglycerides, and fibrinogen in one model and chronic kidney disease (CKD; eGFR < 60) in another. We also examined whether associations of sphingolipids with incident CVD differed by age, sex, BMI, CKD, or duration of diabetes by adding product interaction terms to the multivariable models. To account for multiple comparisons in these sensitivity analyses, we used a significance threshold of p < 0.001 (8 sphingolipid species and 6 additional models; p < 0.05/48).

Inverse-variance-weighted fixed effects meta-analyses were conducted to combine results from the two cohorts using the log hazard ratios (SHFS), log odds ratios (SHS), and their corresponding standard errors in STATA 16.0 (Stata Corporation, College Station, TX); for the purposes of this manuscript, we refer to resulting meta-analyzed parameter as “relative risk.” Inverse-variance-weighted fixed-effects meta-analyses approximate results that would be obtained if the data from all studies could be analyzed together with adjustment for study [25]. Heterogeneity was assessed via I^2^ variance [26].

Multiple imputation with chained equations was used to impute missing values for smoking (SHFS: n = 2; SHS: n = 9), alcohol consumption (SHFS: n = 30; SHS: n = 5), education (SHFS: n = 9), BMI (SHFS: n = 7), waist circumference (SHFS: n = 4), LDL-cholesterol (SHFS: n = 10; SHS: n = 18), duration of diabetes (SHFS: n = 32; SHS: n = 1), physical activity (SHFS: n = 206; SHS: n = 12), systolic blood pressure (n = 1), fibrinogen (SHFS: n = 3; SHS: n = 3), triglycerides (SHFS: n = 3; SHS: n = 1), HDL-cholesterol (SHFS: n = 7; SHS: n = 2), and CKD (SHFS: n = 2; SHS: n = 4) using information on age, sex, site, treated hypertension, treated hyperlipidemia, and type of diabetes treatment [18, 27, 28]. Schoenfeld residuals were evaluated to test the proportional hazards assumption in SHFS Cox models; Martingale residuals were used to assess the linearity of associations; and delta-betas were reviewed to assess the impact of potential influential outliers.

A Bonferroni correction was used to adjust for multiple comparisons; a significance threshold of 0.0063 (0.05/8 sphingolipid species) was used.

## Results

Participant characteristics at the time of their sphingolipid measurement are listed in Table 1. The average age of participants was 49 years (range: 15–86 years) in SHFS, 61 years (range: 49–78 years) in SHS controls, and 62 years (range 49–79 years) in SHS cases; 62% and 64% of SHFS and SHS participants, respectively, were women. On average, participants were diagnosed with diabetes 7 years before their sphingolipid measurement. SHFS participants had larger BMIs and waist circumferences, lower LDL-cholesterol, a lower prevalence of CKD, and a higher prevalence of treated hypertension and treated hyperlipidemia than SHS participants, despite being 12 years younger, on average. Smoking (current or past) was more common in SHS cases and controls, while current alcohol use was more common among SHFS participants.

**Table 1:** Baseline characteristics of SHFS and SHS participants

	SHFS (n = 597)		SHS controls (n = 189)		SHS cases (n = 78)	
	Mean or %	SD	Mean or %	SD	Mean or %	SD
Age, years	49	14	61	8	62	8
Education, years	12	2	11	3	11	9
Smoking, %						
Current	33%		37%		35%	
Past	30%		39%		37%	
Alcohol consumption, %						
Current	46%		31%		22%	
Past	41%		47%		55%	
Physical activity^a^	4299	3260	75	76	77	16
BMI, kg/m^2^	36	8	31	5	32	17
Waist circumference, cm	115	18	107	11	110	18
LDL-cholesterol, mg/dL	101	31	123	31	127	19
HDL-cholesterol, mg/dL	47	14	37	10	36	20
Triglycerides, mg/dL	242	354	190	149	209	21
Systolic blood pressure, mmHg	128	18	127	18	136	22
Treated hypertension, %	49%		33%		45%	
Duration of diabetes, years	6	8	7	8	11	24
Diabetes medication, %						
Insulin	10%		19%		27%	
Oral	46%		37%		33%	
Both	9%		2%		10%	
Neither	35%		43%		29%	
Prevalent CKD, %	9%		16%		32%	
Treated lipidemia, %	12%		1%		1%	

^a^Physical activity was defined as pedometer measured steps per day in SHFS and metabolic equivalent task hours per week in SHS

Concentrations of each of the measured sphingolipids for each cohort are presented in Table 2. Overall, the distributions of each sphingolipid were similar in the two cohorts, and the concentration at the 90th percentile was roughly double that of concentration at the 10th percentile for each sphingolipid. Correlations of the measured sphingolipids are presented in Additional file 1: Fig. S1.

**Table 2:** SHFS and SHS participant sphingolipid concentrations (µM)

	SHFS (n = 597)				SHS controls (n = 189)				SHS cases (n = 78)			
	Mean	SD	Range	90th/10th pct	Mean	SD	Range	90th/10th pct	Mean	SD	Range	90th/10th pct
Cer-16	0.20	0.07	0.06–0.58	2.2	0.32	0.08	0.13–0.64	1.9	0.33	0.09	0.16–0.6	2.2
Cer-20	0.07	0.03	0.01–0.29	2.8	0.11	0.04	0.04–0.38	2.5	0.11	0.04	0.06–0.27	2.2
Cer-22	0.81	0.33	0.23–3.16	2.5	0.76	0.22	0.34–1.84	1.9	0.74	0.22	0.29–1.43	2.1
Cer-24	5.62	2.06	1.75–18.96	2.4	5.15	1.36	2.33–9.94	1.9	5.06	1.41	2.23–9.93	1.9
SM-16	135.21	28.73	59.79–275.51	1.6	131.37	18.38	84.36–186.86	1.4	134.74	25.97	76.13–226.01	1.6
SM-20	20.33	5.00	8.76–44.53	1.9	15.70	3.17	9.17–23.97	1.8	15.15	3.49	6.65–28.98	1.7
SM-22	35.82	9.40	13.09–76.09	1.9	26.21	5.52	14.03–39.15	1.8	25.23	6.06	12.41–48.77	1.8
SM-24	19.36	5.45	7.35–44.67	2.0	14.06	3.36	7.3–23.76	1.8	13.42	3.42	6.46–24.12	1.9

Over a median 14 years of follow-up (range: 0–16 years), we identified 116 cases of incident CVD among the 597 SHFS participants with diabetes. These included 39 incident MI events, 16 strokes (12 ischemic; 3 hemorrhagic, 1 fatal/undetermined), 99 incident total cases of ASCVD, and 33 incident cases of HF (outcomes not mutually exclusive). Among the 78 SHS cases, the median time until incident CVD was 3.8 years; these included 17 MI events, 11 strokes (7 ischemic, 2 hemorrhagic, 2 fatal/undetermined), 50 total cases of incident ASCVD, and 17 cases of incident HF.

**Fig. 1:**
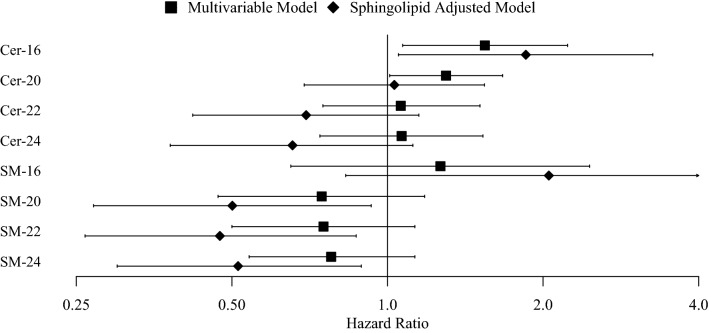
Meta-analyzed adjusted risk of incident CVD in SHFS and SHS. Multivariable Model includes terms for age, sex, study site (in SHFS only), education, smoking, physical activity, BMI, waist circumference, LDL-cholesterol, systolic blood pressure, treated hypertension, duration of diabetes, type of diabetes medication used, and treated hyperlipidemia. Sphingolipid Adjusted Model further adjusts for one of the other species: Cer-16 and SM-16 models include adjustment for Cer-22 and SM-22, respectively; Cer-20, -22, -24, and SM-20, -22, and -24 models include adjustment for Cer-16 and SM-16, respectively

Meta-analyzed results from the SHFS cohort and from the case–control study nested in SHS that evaluate the associations between sphingolipids and risk of incident CVD among participants with prevalent diabetes are presented in Table 3; Fig. 1 (and Additional file 2: Tables S1 and S2). Although we could not demonstrate that any of the investigated sphingolipids were associated with incident CVD at our pre-specified Bonferroni threshold of 0.0063, there is evidence to suggest that Cer-16 is associated with elevated CVD risk (per two-fold increase in Cer16—hazard ratio (HR): 1.54; 95% confidence interval (CI) 1.07–2.23; p-value: 0.021). After adjustment for another sphingolipid species, Cer-16 was associated with increased risk, while SM-20, -22, -24, were associated with decreased risk of incident CVD at the 0.05 significance level.

**Table 3:** Meta-analyzed adjusted risk of incident cardiovascular disease in SHFS and SHS

	Model 1			Model 2			Model 3		
	RR	95% CI	p-value	RR	95% CI	p-value	RR	95% CI	p-value
Cer-16	1.71	(1.18, 2.48)	0.005	1.54	(1.07, 2.23)	0.021	1.85	(1.05, 3.25)	0.033
Cer-20	1.27	(0.98, 1.65)	0.072	1.30	(1.01, 1.68)	0.044	1.03	(0.69, 1.54)	0.876
Cer-22	1.09	(0.76, 1.57)	0.640	1.06	(0.75, 1.5)	0.741	0.70	(0.42, 1.16)	0.162
Cer-24	1.13	(0.77, 1.64)	0.534	1.06	(0.74, 1.52)	0.738	0.65	(0.38, 1.12)	0.122
SM-16	1.62	(0.84, 3.14)	0.154	1.26	(0.65, 2.46)	0.492	2.04	(0.82, 5.05)	0.125
SM-20	0.80	(0.52, 1.24)	0.328	0.75	(0.48, 1.18)	0.209	0.50	(0.27, 0.93)	0.028
SM-22	0.85	(0.57, 1.27)	0.414	0.75	(0.51, 1.13)	0.169	0.48	(0.26, 0.87)	0.016
SM-24	0.88	(0.61, 1.25)	0.470	0.78	(0.54, 1.14)	0.198	0.52	(0.3, 0.89)	0.018

Model 1 includes terms for age, sex, and study site (SHFS only). Model 2 additionally includes terms for education, smoking, physical activity, BMI, waist circumference, LDL-cholesterol, systolic blood pressure, treated hypertension, duration of diabetes, type of diabetes medication used, and treated hyperlipidemia. Model 3 further adjusts for one of the other species: Cer-16 and SM-16 models include adjustment for Cer-22 and SM-22, respectively; Cer-20, -22, -24, and SM-20, -22, and -24 models include adjustment for Cer-16 and SM-16, respectively

Results from models that adjusted for HDL-cholesterol, triglycerides, and fibrinogen and CKD were similar to our primary analysis (Additional file 2: Table S3). There was no evidence of interactions of any of the metabolites with age, sex, BMI, CKD, or duration of diabetes.

There was no evidence of significant heterogeneity in the multivariable model between the two cohorts, and differences between the cohorts had little effect on the associations of Cer-16, Cer-22, Cer-24, SM-16, and SM-18 (I^2^ < 25%) and a moderate effect on the associations of Cer-20, SM-22, and SM-24 (I^2^ < 50%). The proportional hazards assumption was not violated in any SHFS models, and there was no evidence of departure from linearity in any of the primary models.

Results from Cox regression models of sphingolipids with incident ASCVD and heart failure in SHFS are presented in Table 4. In our multivariable model, two of the investigated sphingolipids, Cer-16 and Cer-20, were associated with greater risk of incident ASCVD (Cer-16 HR: 2.01, 95% CI 1.35, 2.99, p-value: 0.0006; Cer-20 HR: 1.70, 95% CI 1.32, 2.19, p-value: < 0.0001). After adjustment for other sphingolipids, neither sphingolipid met the Bonferroni threshold for significance, but there was evidence to suggest that Cer-16 was associated with higher risk of ASCVD (HR: 2.33, 95% CI 1.14, 4.77, p-value: 0.03), while the association with Cer-20 was attenuated (HR: 1.29, 95% CI 0.74, 2.25, p-value: 0.22). We did not observe any significant associations of the investigated sphingolipids with incident heart failure.

**Table 4:** Adjusted risk of incident atherosclerotic cardiovascular disease and heart failure in SHFS

	Atherosclerotic cardiovascular disease						Heart failure					
	Multivariable model			Sphingolipid adjusted model			Multivariable model			Sphingolipid adjusted model		
	HR	95% CI	p	HR	95% CI	p	HR	95% CI	p	HR	95% CI	p
Cer-16	2.01	(1.35, 2.99)	0.0006	2.26	(1.09, 4.66)	0.0276	1.77	(0.73, 4.28)	0.21	2.57	(0.76, 8.66)	0.13
Cer-20	1.7	(1.32, 2.19)	0.00004	1.42	(0.81, 2.47)	0.2180	1.24	(0.64, 2.39)	0.52	0.83	(0.32, 2.18)	0.71
Cer-22	1.44	(0.96, 2.17)	0.08	0.88	(0.44, 1.75)	0.7133	1.22	(0.59, 2.52)	0.59	0.67	(0.26, 1.72)	0.41
Cer-24	1.36	(0.86, 2.14)	0.19	0.72	(0.35, 1.48)	0.3705	1.07	(0.45, 2.58)	0.87	0.45	(0.14, 1.44)	0.18
SM-16	2.07	(0.92, 4.67)	0.08	2.97	(0.96, 9.18)	0.0588	0.98	(0.28, 3.50)	0.98	0.69	(0.13, 3.74)	0.67
SM-20	1.09	(0.62, 1.91)	0.76	0.61	(0.27, 1.37)	0.2343	1.14	(0.46, 2.83)	0.78	1.27	(0.38, 4.22)	0.69
SM-22	1.08	(0.64, 1.82)	0.78	0.6	(0.28, 1.31)	0.2031	1.28	(0.46, 3.57)	0.63	1.59	(0.41, 6.10)	0.50
SM-24	1.03	(0.63, 1.70)	0.91	0.61	(0.30, 1.24)	0.1709	1.28	(0.49, 3.34)	0.62	1.52	(0.49, 4.77)	0.47

Number of incident events: Atherosclerotic Heart Disease (n = 99); HF (n = 33). Models are adjusted for age, sex, study site, education, smoking, physical activity, BMI, waist circumference, LDL-cholesterol, systolic blood pressure, treated hypertension, duration of diabetes, type of diabetes medication used, and treated hyperlipidemia; for the sphingolipid adjusted models, Cer-16 and SM-16 models include additional adjustment for Cer-22 and SM-22, respectively; Cer-20, -22, -24, and SM-20, -22, and -24 models include additional adjustment for Cer-16 and SM-16, respectively

## Discussion

Among 597 SHFS and 267 SH study participants with prevalent diabetes, we did not detect statistically significant associations of the investigated sphingolipids with our composite CVD outcome at our pre-specified significance threshold. However, in agreement with our stated hypothesis, we observed an association of Cer-16 with higher risk of CVD, and in analyses adjusted for SM-16, associations of SM-20, 22 and 24 with lower risk of CVD. Further, in secondary analyses, we report that Cer-16 and possibly Cer-20 are associated with increased risk of incident atherosclerotic CVD. Although previous studies have reported associations of specific ceramides with both CVD and diabetes independently, to our knowledge, this is the first prospective study to investigate associations of ceramide and sphingomyelin species with CVD among adults with type 2 diabetes.

An association of Cer-16 with higher risk of CVD has been previously reported. In particular, Cer-16 was associated with incident risk of major cardiovascular events in the FINRISK and EPIC-Potsdam studies [29, 30]. In addition, in a recent meta-analysis that pooled results of cohort studies of incident and recurrent CVD, Cer-16 was associated with CVD risk [15]. Our study attempts to extend these findings by exploring these associations in a different ethnicity and among high-risk adults with type 2 diabetes. Further, we previously reported that circulating ceramides are associated with higher risk of incident diabetes in the same population, as well as associate with early markers of diabetes [10, 18, 19]. The current study now suggests that ceramides, in particular Cer-16, not only are associated with higher risk of incident diabetes, but also higher risk of incident CVD after diabetes is diagnosed. In addition, the studies of circulating ceramides and CVD mentioned above did not examine sphingomyelins. A recent publication from the Da Qing Diabetes Study did not investigate sphingomyelins with a very long chain saturated fatty acid, but did report an association of SM-16 with higher CVD risk among people with diabetes, which is suggested (but not significant) in our findings [31]. Our study suggests for the first time that sphingomyelins with a very long chain saturated fatty acid are associated with lower risk of incident CVD among adults with type 2 diabetes.

As is common in longitudinal population-based studies, our CVD outcome was a composite of different cardiovascular events. Because 85% of the events of our study were either MI, CHD, or ischemic stroke, we examined the sphingolipid associations with the composite outcome of “atherosclerotic CVD” in secondary analyses. Circulating Cer-16 and Cer-20 were associated with higher risk of incident atherosclerotic CVD; however, these two ceramides are highly correlated (correlation = 0.76 in SHFS, 0.69 in SHS) and the association of Cer-20 was greatly diminished by adjustment for Cer-16, suggesting that Cer-16 is the primary species associated with risk. There is less support in the literature for an association of Cer-16 with atherosclerotic outcomes in cohort studies. An association of the ratio Cer-24/Cer-16 with lower risk of incident CHD has been reported in the Framingham Heart Study and the Study of Health in Pomerania; however, the CHD association was entirely due to Cer-24, and Cer-16 itself was not associated with CHD risk in both cohorts, with point estimates close to the null [8, 18, 24]. Further studies will be needed to replicate the association of Cer-16 with atherosclerotic outcomes.

We did not observe an association of ceramides and sphingomyelins with heart failure. This is in contrast with findings in the Cardiovascular Health Study where Cer-16 and SM-16 are associated with increased risk of HF while longer-chain Cer and SM are associated with decreased risk [16]. Given the small number of heart failure events and limited power to find HF associations in this current study, the apparent lack of association should be interpreted with caution.

Ceramides and sphingomyelins may play a direct role in cardiovascular disease through an effect on atherosclerosis and inflammation, which would be especially influential among people with diabetes [32–34]. Conversion of sphingomyelin in LDL-cholesterol particles to ceramide by sphingomyelinase promotes ceramide-ceramide aggregation, a possible early event in atherosclerosis, and lowered plasma sphingomyelin levels in mice resulted in reduced atherosclerosis [35–37]. In addition to their possible role in atherosclerosis, ceramides are implicated in reperfusion injury, lipotoxicity and apoptosis, biological activities that may influence the risks of heart failure and arrhythmia [32, 34, 38].

Strengths of this study include participants with diabetes from multi-site prospective studies of AIs—a population that itself suffers a relatively high prevalence of diabetes complications; detailed and thorough assessment of participant characteristics and potential confounders; and objective laboratory measurements of ceramide and sphingomyelin levels. This study also has several limitations. We used a composite measure of CVD as the primary outcome in this analysis, and the limited number of events of MI and stroke did not allow separate analyses of these outcomes. In SHFS we were able to categorize events as either ASCVD or HF, but due to a limited number of events in SH and the case–control sampling we were not able to make the same classification in this cohort and hence could not meta-analyze the CVD subtypes. Further, in the SH study, cases and controls were not matched on site. As a result, we were not able to adjust the SH analysis for site; since diet and lifestyle patterns differ between the three SH sites, SH estimates may be biased due to unmeasured confounding.

In conclusion, our findings in this population-based longitudinal study of AIs with type 2 diabetes suggest that several ceramide and sphingomyelin species may be associated with incident CVD, particularly with ASCVD. Although these associations did not meet our pre-specified threshold for statistical significance, our findings for Cer-16 add credence to recent findings in other cohorts, and our findings for SM with a very long chain saturated fatty acid encourage further research in other populations.

## Supplementary Information

**Additional file 1: Figure S1.** Correlation of Sphingolipid Species in SHFS and SHS.

**Additional file 2: Table S1.** Risk of incident CVD per two-fold higher sphingolipid level in SHFS. **Table S2.** Odds of incident CVD per two-fold higher sphingolipid level in SHS. **Table S3.** Sensitivity analysis—associations of sphingolipids with incident CVD risk after adjustment for HDL and Triglycerides, Fibrinogen, and Chronic Kidney Disease.

## Data Availability

Due to privacy agreements with the tribal communities involved in this study, access to study data is restricted. Further information can be found at https://strongheartstudy.org/.

## Abbreviations

CVD: Cardiovascular disease
Cer: Ceramide
SM: Sphingomyelin
AI: American Indian
SHS: Strong Heart Study
SHFS: Strong Heart Family Study
MI: Myocardial infarction
ASCVD: Atherosclerotic cardiovascular disease
HF: Heart failure
CHD: Coronary heart disease
BMI: Body mass index
LDL: Low density lipoprotein-cholesterol
HDL: High density lipoprotein-cholesterol
eGFR: Estimated glomerular filtration rate
CKD: Chronic kidney disease
HR: Hazard ratio
CI: Confidence interval
