# Supplementary figures and images for: Circulating ceramides and sphingomyelins and the risk of incident cardiovascular disease among people with diabetes: the strong heart study
Source: Cardiovasc Diabetol. 2022 Aug 30;21:167. doi: 10.1186/s12933-022-01596-4 (PMC9429431; doi:10.1186/s12933-022-01596-4)

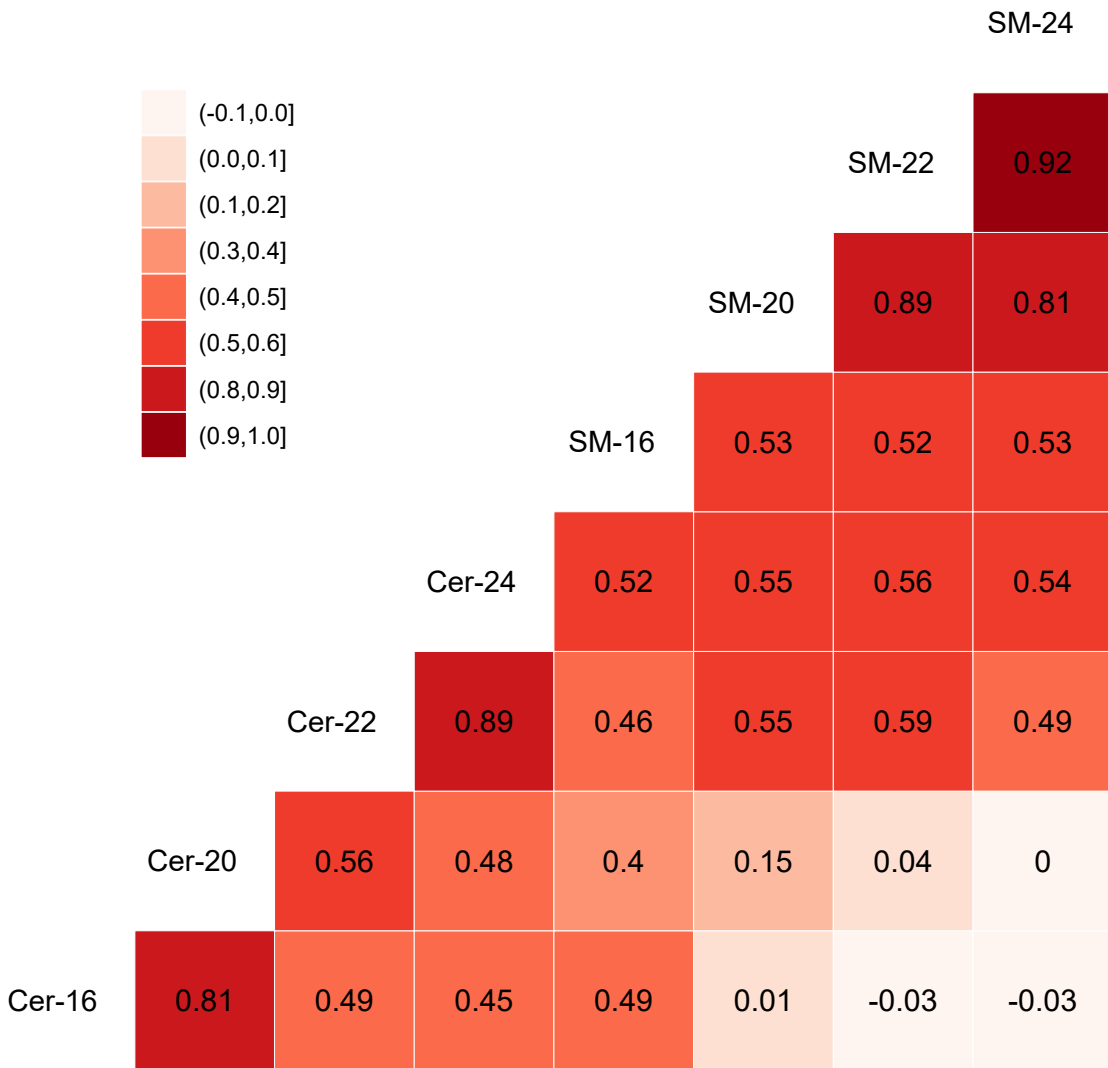

Supplement: Supplementary file 1 — Additional file 1: Figure S1. Correlation of Sphingolipid Species in SHFS and SHS. [file 12933_2022_1596_MOESM1_ESM.pdf]
