# Supplementary material for: Circulating ceramides and sphingomyelins and the risk of incident cardiovascular disease among people with diabetes: the strong heart study
Source: Cardiovasc Diabetol. 2022 Aug 30;21:167. doi: 10.1186/s12933-022-01596-4 (PMC9429431; doi:10.1186/s12933-022-01596-4)
Supplement: Supplementary file 2 — Additional file 2: Table S1. Risk of incident CVD per two-fold higher sphingolipid level in SHFS. Table S2. Odds of incident CVD per two-fold higher sphingolipid level in SHS. Table S3. Sensitivity analysis—associations of sphingolipids with incident CVD risk after adjustment for HDL and Triglycerides, Fibrinogen, and Chronic Kidney Disease. [file 12933_2022_1596_MOESM2_ESM.pdf]

Table S1. Risk of incident CVD per two-fold higher sphingolipid level in SHFS

	Model 1			Model 2			Model 3		
	HR	95% CI	p	HR	95% CI	p	HR	95% CI	p
Cer-16	1.90	(1.24, 2.91)	0.003	1.83	(1.22, 2.76)	0.004	1.78	(1.17, 2.72)	0.007
Cer-20	1.41	(1.04, 1.91)	0.025	1.49	(1.12, 1.98)	0.006	1.46	(1.08, 1.95)	0.012
Cer-22	1.24	(0.81, 1.89)	0.328	1.27	(0.85, 1.89)	0.237	1.20	(0.80, 1.81)	0.382
Cer-24	1.26	(0.81, 1.94)	0.303	1.22	(0.82, 1.82)	0.325	1.15	(0.75, 1.75)	0.530
SM-16	1.63	(0.77, 3.47)	0.205	1.54	(0.74, 3.20)	0.252	1.55	(0.81, 2.99)	0.189
SM-20	0.91	(0.55, 1.50)	0.708	0.89	(0.54, 1.45)	0.631	0.88	(0.55, 1.40)	0.586
SM-22	0.95	(0.60, 1.49)	0.821	0.88	(0.57, 1.36)	0.580	0.89	(0.59, 1.33)	0.566
SM-24	0.98	(0.66, 1.46)	0.926	0.90	(0.60, 1.35)	0.614	0.92	(0.63, 1.34)	0.653

Model 1 includes terms for age, sex, and study site (but site only in SHFS!). Model 2 additionally includes terms for education, smoking, physical activity, BMI, waist circumference, LDL-cholesterol, systolic blood pressure, treated hypertension, duration of diabetes, type of diabetes medication used, and treated hyperlipidemia. Model 3 further adjusts for one of the other species: Cer-16 and SM-16 models include adjustment for Cer-22 and SM-22, respectively; Cer-20, -22, -24, and SM-20, -22, and -24 models include adjustment for Cer-16 and SM-16, respectively.

Table S2. Odds of incident CVD per two-fold higher sphingolipid level in SHS

	Model 1			Model 2			Model 3		
	HR	95% CI	p	HR	95% CI	p	HR	95% CI	p
Cer-16	1.22	(0.57, 2.62)	0.615	0.71	(0.30, 1.69)	0.441	0.65	(0.26, 1.60)	0.345
Cer-20	0.93	(0.55, 1.56)	0.780	0.71	(0.40, 1.28)	0.259	0.66	(0.36, 1.22)	0.188
Cer-22	0.79	(0.40, 1.56)	0.494	0.57	(0.27, 1.18)	0.131	0.44	(0.20, 0.98)	0.043
Cer-24	0.81	(0.38, 1.72)	0.585	0.59	(0.26, 1.34)	0.206	0.49	(0.21, 1.16)	0.105
SM-16	1.58	(0.40, 6.18)	0.513	0.51	(0.11, 2.47)	0.406	0.68	(0.12, 3.70)	0.652
SM-20	0.54	(0.22, 1.33)	0.179	0.32	(0.11, 0.97)	0.045	0.35	(0.11, 1.11)	0.074
SM-22	0.55	(0.23, 1.32)	0.182	0.28	(0.10, 0.82)	0.021	0.29	(0.09, 0.91)	0.033
SM-24	0.56	(0.25, 1.24)	0.153	0.34	(0.13, 0.91)	0.031	0.36	(0.13, 1.03)	0.057

Model 1 includes terms for age, sex, and study site. Model 2 additionally includes terms for education, smoking, physical activity, BMI, waist circumference, LDL-cholesterol, systolic blood pressure, treated hypertension, duration of diabetes, type of diabetes medication used, and treated hyperlipidemia. Model 3 further adjusts for one of the other species: Cer-16 and SM-16 models include adjustment for Cer-22 and SM-22, respectively; Cer-20, -22, -24, and SM-20, -22, and -24 models include adjustment for Cer-16 and SM-16, respectively.

Table S3. Sensitivity analysis – Associations of sphingolipids with incident CVD risk after adjustment for HDL and Triglycerides, Fibrinogen, and Chronic Kidney Disease

				Additional Adjustments								
	Primary Model			HDL and Triglycerides			Fibrinogen			CKD		
	RR	95% CI	p-value	Risk	95% CI	p-value	Risk	95% CI	p-value	Risk	95% CI	p-value
Cer-16	1.54	(1.07, 2.23)	0.021	1.49	(1.01, 2.18)	0.04	1.482	(1.03, 2.13)	0.035	1.552	(1.07, 2.26)	0.021
Cer-20	1.30	(1.01, 1.68)	0.044	1.26	(0.96, 1.64)	0.09	1.276	(0.99, 1.64)	0.059	1.295	(1, 1.67)	0.047
Cer-22	1.06	(0.75, 1.5)	0.741	0.97	(0.68, 1.4)	0.89	1.07	(0.76, 1.51)	0.703	1.068	(0.75, 1.52)	0.713
Cer-24	1.06	(0.74, 1.52)	0.738	0.97	(0.66, 1.42)	0.88	1.078	(0.75, 1.54)	0.682	1.081	(0.75, 1.56)	0.675
SM-16	1.26	(0.65, 2.46)	0.492	1.39	(0.76, 2.57)	0.29	1.169	(0.6, 2.29)	0.649	1.267	(0.65, 2.46)	0.484
SM-20	0.75	(0.48, 1.18)	0.209	0.77	(0.5, 1.19)	0.25	0.759	(0.48, 1.19)	0.231	0.746	(0.47, 1.18)	0.208
SM-22	0.75	(0.51, 1.13)	0.169	0.78	(0.53, 1.15)	0.21	0.776	(0.51, 1.17)	0.226	0.746	(0.5, 1.12)	0.16
SM-24	0.78	(0.54, 1.14)	0.198	0.82	(0.58, 1.18)	0.29	0.819	(0.56, 1.21)	0.315	0.772	(0.53, 1.13)	0.18

Models are adjusted for age, sex, study site, education, smoking, physical activity, BMI, waist circumference, LDL-cholesterol, systolic blood pressure, treated hypertension, duration of diabetes, type of diabetes medication used, and treated hyperlipidemia
